# Characterization of full-length sequenced cDNA inserts (FLIcs) from Atlantic salmon (*Salmo salar*)

**DOI:** 10.1186/1471-2164-10-502

**Published:** 2009-10-30

**Authors:** Rune Andreassen, Sigbjørn Lunner, Bjørn Høyheim

**Affiliations:** 1 BasAM-Genetics, Norwegian School of Veterinary Science, PO Box 8146 DEP, NO-0033 Oslo, Norway; 2 CIGENE-Centre for Integrative Genetics, Ås, Norway; 3 Faculty of Health Sciences, Oslo University College, Oslo, Norway

## Abstract

**Background:**

Sequencing of the Atlantic salmon genome is now being planned by an international research consortium. Full-length sequenced inserts from cDNAs (FLIcs) are an important tool for correct annotation and clustering of the genomic sequence in any species. The large amount of highly similar duplicate sequences caused by the relatively recent genome duplication in the salmonid ancestor represents a particular challenge for the genome project. FLIcs will therefore be an extremely useful resource for the Atlantic salmon sequencing project. In addition to be helpful in order to distinguish between duplicate genome regions and in determining correct gene structures, FLIcs are an important resource for functional genomic studies and for investigation of regulatory elements controlling gene expression. In contrast to the large number of ESTs available, including the ESTs from 23 developmental and tissue specific cDNA libraries contributed by the Salmon Genome Project (SGP), the number of sequences where the full-length of the cDNA insert has been determined has been small.

**Results:**

High quality full-length insert sequences from 560 pre-smolt white muscle tissue specific cDNAs were generated, accession numbers [GenBank: BT043497 - BT044056]. Five hundred and ten (91%) of the transcripts were annotated using Gene Ontology (GO) terms and 440 of the FLIcs are likely to contain a complete coding sequence (cCDS). The sequence information was used to identify putative paralogs, characterize salmon Kozak motifs, polyadenylation signal variation and to identify motifs likely to be involved in the regulation of particular genes. Finally, conserved 7-mers in the 3'UTRs were identified, of which some were identical to miRNA target sequences.

**Conclusion:**

This paper describes the first Atlantic salmon FLIcs from a tissue and developmental stage specific cDNA library. We have demonstrated that many FLIcs contained a complete coding sequence (cCDS). This suggests that the remaining cDNA libraries generated by SGP represent a valuable cCDS FLIc source. The conservation of 7-mers in 3'UTRs indicates that these motifs are functionally important. Identity between some of these 7-mers and miRNA target sequences suggests that they are miRNA targets in *Salmo salar *transcripts as well.

## Background

Atlantic salmon (*Salmo salar*) is an important aquaculture species, and there is also a considerable commercial harvesting of wild salmon. As a consequence of the economic interest in salmon, various genomic resources have been developed to identify genes and genomic mechanisms responsible for commercially important traits. These resources include a BAC library, the corresponding physical map and several linkage maps [1-5]. In addition several cDNA libraries have been constructed [6-10] and at present 494,094 ESTs have been submitted to GenBank [11].

The salmon genome is complex due to a relatively recent genome duplication believed to have occurred between 25 and 120 million years ago in the common salmonid ancestor [12]. Analysis of segregation ratios in salmonids has revealed disomic inheritance in females while there is a mixture of disomic and tetrasomic inheritance in males [12,13]. Divergence of loci duplicated via tetraploidy is depending on complete reestablishment of disomic inheritance, and present salmonids appear to have retained more than 50% of loci as duplicates [14]. This suggests that salmonids are in the process of re-establishing disomic inheritance. Studies of the salmon genome might therefore contribute important biological knowledge on the evolution of ohnologs (duplicate sequences that originate from a whole genome duplication) [15].

Transcript sequences that represent the coding regions of genes may be predicted based on consensus assemblies of overlapping ESTs such as the gene indices in TIGR database [16]. These clusters of tentative consensus sequences (TCs) serve as a valuable resource for putative gene products. However, these reconstructions are prone to error caused by low quality of single-pass sequences, alternative splice forms, expressed pseudogenes and sequence similarities between transcripts within gene families. One would expect that the large number of almost identical ohnologous sequences would make such gene transcript predictions particularly challenging in salmonid species. In agreement with this, results from studies using salmon EST data as a source for SNP discovery show that when clustering EST-sequences into consensus sequences there is a high frequency of "SNPs" with heterozygote excess. This indicates that a large amount of the ESTs in such clusters are derived from different loci [5,17].

The most useful transcript sequences are derived from high quality full-length sequencing of inserts from cDNA clones (FLIcs) that contain the complete protein coding sequence (cCDS). By determining the cCDS from one single clone the errors caused by incorrect clustering of non-allelic sequences are omitted. High quality sequences based on multi-pass reads of the CDS from FLIcs are therefore the most reliable source for transcript prediction. In addition to representing the most suitable mean to predict protein sequences, the data from FLIcs might also be used to identify splice variants as well as to differ between closely related paralogs. Complete CDS FLIcs are also important in genome clustering and annotation. Genomic sequencing of Atlantic salmon is being organised by an international consortium and due to the problems related to the recent duplication of the salmon genome clustering and annotation of the sequence might prove difficult. Thus, a large number of high quality cCDS FLIcs would therefore be of great value in a salmon genome sequencing project. Finally, in full-length insert sequences, where the boundaries of the coding sequences are defined, the additional transcript sequences provide sequence information from 5' and 3'UTRs. Within these non-coding mRNA segments there are sequence motifs that are important in regulation of gene expression. Access to reliable sequence information from UTRs is a precondition to identify such functional motifs. Together, the above mentioned use of cCDS FLIcs and their source cDNA clones has led to large scale sequencing of full-length inserts in several species [18-23]. Despite the apparent usefulness of cCDS FLIcs few salmon FLIcs were available in public databases at the time this study was initiated.

The aim of this study has been to determine the full sequence of the inserts in a set of selected Atlantic salmon cDNA clones to provide a larger amount of high quality sequenced transcripts with complete CDSs from a single tissue and developmental stage. Clones were selected from a white muscle tissue specific library from pre-smolt developmental stage since our research at the time this study was initiated also focused on discovering genes that might be important to the ability of depositing dietary cartenoid pigments in muscle tissue. The results from the annotation of full-length sequenced inserts and identification of cDNA transcripts likely to contain complete CDSs are presented. We also describe some general characteristics of *Salmo salar *transcripts such as the Kozak consensus sequence, polyadenylation signal variation and we identify conserved, putatively functional, elements in the UTRs.

## Results and Discussion

### Full- length insert cDNA sequences and gene ontology annotation

The cDNA clones chosen for full-length insert sequencing were from a white muscle and pre-smolt developmental stage specific library. The library consisted originally of 6,656 ESTs that were assembled in 567 contigs and 1,091 singlets [10]. One representative clone from each of the contigs as well as all singlets was initially included in our material. The 5' single pass sequences from the selected cDNA clones were then used as queries in similarity searches against publicly available databases (see methods). The purpose of this exercise was to remove cDNA clones not likely to contain inserts with a complete coding sequence. The exercise reduced the number of clones with putative cCDSs to 560 (273 selected from contigs and 287 singlets). The full-length inserts (FLIcs) from these cDNA clones were sequenced using a primer walking strategy. Manual inspection of all assembled sequences was performed to ensure a high quality on the consensus sequence data from each of the FLIcs. All sequences within the FLIcs likely to represent coding sequences (see below) were covered by at least two reads. The average number of reads (total number of bases sequenced/total number of bases of all FLIcs) in the finished sequences was 4.1. The FLIc consensus sequences from the 560 cDNA clones were submitted to GenBank Aug. 20^th^, 2008 [GenBank: BT043497 - BT044056].

To measure the success rate of our selection procedure we used a homology search based approach to estimate the number of FLIcs in our material that was likely to contain complete coding sequences (cCDS FLIcs). We performed these similarity searches with BLASTX against the RefSeq database [24] using each of the FLIc consensus sequences as input. Input sequences that returned matches with expectation values (E-values) less than 10^-15 ^and positives score of at least 60% was further evaluated by manual inspection of the pairwise alignments. If the ORF from the FLIc sequence revealed a startcodon and a stopcodon in agreement with the match in the RefSeq database, the FLIc was considered to contain a cCDS. Based on these criteria there were 440 transcripts with complete coding sequences (cCDS FLIcs) in our material, indicating a success rate of about 79%. Accession numbers to the cCDS FLIcs are [GenBank: BT043497-BT043935 and BT043946]. The E-values returned from the alignments of those transcripts that we classified as cCDS FLIcs were in most cases (>98%) below 10^-30^. Some cCDS FLIcs with short coding sequences (e.g. transcripts annotated as ribosomal protein mRNA) returned slightly higher E-values. All these alignments revealed positive scores above 80% indicating that the slightly higher E-values were caused by a smaller size of the coding sequences rather than a poor match. A summary of cCDS FLIc data is given in table 1.

**Table 1:** Summary of complete CDS FLIcs (cCDS FLIcs)

Number of cCDS FLIcs	440
Average length (bp)^1^	1322
Average length (bp)^2^	1450
Size range (bp) of cCDS FLIcs	282-3791
Success rate^3^	0.79

^1^Average length of all cCDS FLIcs, ^2^Average length of cCDS FLIcs

when transcripts annotated as ribosomal genes (n = 73) were subtracted

^3^Number of cCDS FLIcs/total number of FLIcs

**Figure 1:**
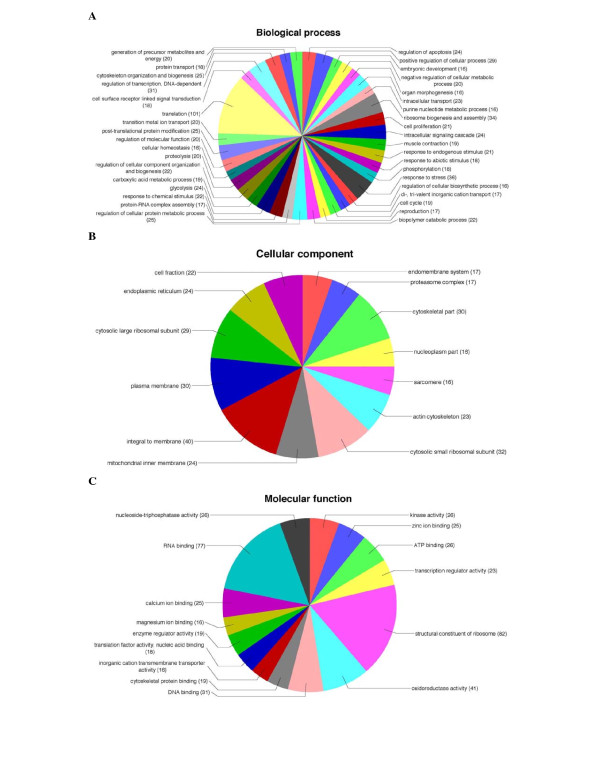
**Distributions of most common GO terms (assigned to 15 or more sequences) in the categories molecular function (1a), biological process (1b) and cellular component (1c)**.

Functional annotation of the 440 cCDS FLIcs was performed according to the Gene Ontology standard [25] using the automatic annotation tool Blast2GO. Blast2GO use an initial BLAST search, the gene ontology terms (GO terms) extracted from hits in the initial homology search and an annotation rule to assign GO terms to the individual query sequences in a dataset [26]. One or more GO terms were assigned to 405 of the cCDS FLIcs. Figure 1a-c shows the most common GO terms assigned to the cCDS FLIcs for each of the categories molecular function, biological process and cellular component. The Blast2GO annotation tool also assigns enzyme codes to the individual sequences, and 199 of the cCDS FLIcs were assigned one or more enzyme codes. The complete dataset with GenBank accession numbers, sequence descriptions, cDNA clone reference numbers, GO terms and enzyme codes assigned to each of the cCDS FLIcs is given in additional file 1.

Hundred and five of the remaining FLIcs are, based on results from the BLASTX analysis, likely to contain partial CDSs of protein coding genes. They revealed E-values below the threshold, but lacked the 5' coding sequence corresponding to the first few amino acids of the matching protein. The putative function of a few of the FLIcs remains unknown. The Blast2GO annotation tool was used to assign GO terms to the FLIcs with partial CDSs. The complete dataset with GenBank accession numbers, sequence descriptions, cDNA clone reference numbers, GO terms and enzyme codes assigned to each of the 105 partial CDS FLIcs is given in additional file 2. The insert sequences from the partial CDS FLIcs lacked some of the 5' coding sequences, but they are high quality sequences and with a mean average FLIc size of 1,450 bp. Each of the sequences are derived from a single clone and thus, they represent more valuable data for transcript prediction than data originating from low quality and short EST sequences.

### Paralogous sequence, splice variants and gene prediction from TCs

Several cCDS FLIcs were, based on sequence similarities in the CDS as well as on GO annotation, likely to be closely related members of gene families (paralogs). There was a total of 54 cases were two or more transcripts were likely to represent paralogs. A summary of these sequences, including GenBank numbers, is given in additional file 3. Some of these FLIcs, e.g. the two transcripts annotated as psmd13 genes [GenBank: BT043856 and BT043857], were highly similar duplicate sequences (98.5% sequence identity). The similarity is, however, lower than expected from allelic sequences [27]. Such highly similar transcripts may represent duplicate sequences originating from the salmonid specific genome duplication. Identification of single locus polymorphisms in this kind of sequences followed by linkage mapping could provide further evidence that these sequences are ohnologs. Sequence analysis of a number of ohnologs could contribute important knowledge on how genes, which originate from genome duplications evolve.

One of the benefits of retrieving mRNA transcript sequence data from cCDS FLIcs is the potential identification of splice variants. Sequence comparisons between cCDS FLIcs in our material suggest that some cCDS FLIcs represent alternatively spliced variants of genes. Table 2 gives an overview of the genes where splice variants were identified. The shorter sequence in each pair revealed a gap of a number of nucleotides when aligned with the other, but apart from that, the pairs were identical, as expected if the shorter transcripts represent splice variants lacking exon sequences that are present in the larger transcripts.

**Table 2:** Pairs of splice variants

**Gene**	**GenBank**	**Clone ID**	**Number of gaps**	**bp size of gap**
nap1l1	BT043845	HM5_0787		
	BT043844	HM5_0277	2	12, 27

troponin T-2	BT043907	HM5_1535		
	BT043904	HM4_2058	1	12
	BT043908	HM6_0331	2	12, 287

troponin T-1	BT043903	HM4_1804		
	BT043909	HM6_0953	1	165

gapdh	BT043825	HM5_2585		
	BT043824	HM4_3976	1	27

Prediction of transcript sequence based on clustering overlapping EST data is potentially more error prone in salmon than most other species given the large amount of almost identical duplicate sequences present in the salmon genome. The cCDS FLIcs offers a first opportunity to evaluate the robustness of such salmon transcript consensus sequences using high quality reference data. In particular, we wanted to examine the degree of erroneous tentative consensus sequences in databases caused by clustering highly similar ESTs originating from different loci. To test this we used the cCDS FLIc data from the group of closely related genes in our material as reference sequences. This material was compared to the tentative consensus sequence (TC) in the DFCI salmon gene index [28] that contained the EST from the same source clone as our reference sequence. A direct comparison of the two sequences could then reveal the amount of sequence difference. Thirty-five such pairwise comparisons were performed (see methods). The mean size of the sequences compared was 1,200 bp. All comparisons that revealed more than four inconsistencies in every 100 bp compared were defined as non-matches. There were eleven such non-matches in the 35 comparisons. Measurements of SNP density in the salmon genome suggest that there is less than one SNP in every 600 bp in introns, and less than one in every 1,400 bp in exons [27,29]. Thus, the amount of sequence difference in the pairs of non-matches was far higher than expected if the references and the TCs were allelic sequences. Some of the sequence differences observed in the pairs defined as non-matches might be explained by the low quality of EST data, however, we do not believe that the sequence differences in non-matches are caused by low quality of the EST data alone. In support of this, a measurement of EST quality by comparing the full-length insert sequence, not to the complete TC, but to the original single pass EST sequence within the TC that where from the FLIc source clone, shows that the errors (a different base in the ESTs when aligned with the FLIc) were less than 1 in every 200 bp. Together this indicate that the TCs defined as non-matches consist of one or more ESTs that are from another locus than that of the FLIc source clone. Approximately one third of the TCs were non-matches when compared to the references. Thus, the results confirm suspicions that highly similar duplicate sequences represent a substantial problem when predicting tentative consensus transcripts from salmon ESTs.

### Kozak motif and polyadenylation signal (PAS) variation in *Salmo salar *transcripts

**Figure 2:**
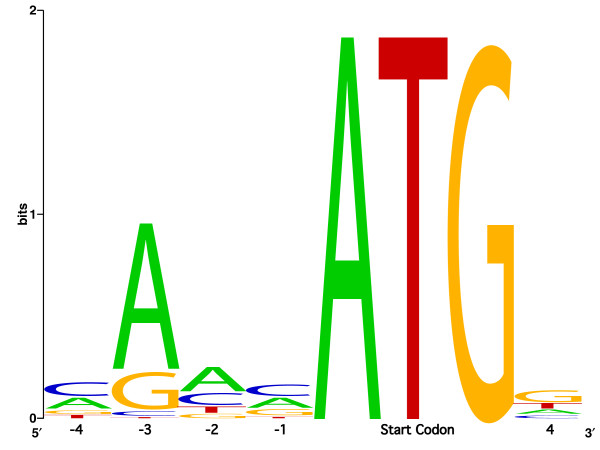
**Kozak consensus sequence surrounding start methionin in the 440 cCDS FLIcs illustrated by use of WebLogo**.

The coding sequences in the cCDS FLIcs define the boundary of the transcriptional unit and the additional FLIc sequences are from the non coding 5' and 3' UTRs. Due to the method used for library construction most of the 5'UTRs are short and incomplete, but nevertheless, most of the transcripts provide some of the sequence immediate upstream of the start of the coding sequence. The AUG start codon context, also referred to as the Kozak sequence, is not strictly conserved in eukaryotes [30,31]. The bases most frequently observed in the *Salmo salar *Kozak motif are CAACATGG. The most conserved bases are, as in other species, position -3 (A/G), the start codon (ATG) and position +4 (G). The frequencies of thymine residues in position -4, -3 and -1 are low, and the frequency of transcripts with either TC or TT in position -4 and -3 is less than 1%. The profile of the *Salmo salar *start codon context spanning the positions -4 to +4 (Kozak motif) is illustrated by use of WebLogo [32] in figure 2.

In 13 cCDS FLIcs the 3'end of the insert sequences terminated in a polyadenylation tail (poly A-tail) of less than 10 adenosine residues. These cases may represent FLIcs with incomplete 3'UTRs due to mispriming from internal sites in the library construction. In the remaining 427 cCDS FLIcs there was a poly A-tail consisting of ten or more consecutive adenosine residues adjacent to the 3' vector sequence. We considered these cCDS FLIcs as transcripts with complete 3'UTRs. The size of 3'UTRs in transcripts annotated as ribosomal mRNA (all less than 80 bp) was considerably shorter than the 3'UTRs in the other transcripts. The average size of the 3'UTR in the cCDS FLIcs, not including ribosomal mRNAs, was 517 bp.

Several studies have shown that different variants of the polyadenylation signal (PAS) exists, and that the frequency distribution of the most common PAS versus alternative less common variants is species, tissue and possibly developmental stage dependent. The choice of which motif is used as PAS might be important in gene regulation, possibly by including or excluding downstream regulatory motifs that are located between such alternative PAS [33-37]. The most common PAS observed immediate upstream of the polyA-tail (within 35 bp) in the tissue and developmental stage specific salmon transcripts from this study was the canonical AAUAAA (348 transcripts, 81%). The second most common variant was AUUAAA present in 50 transcripts (12%). These findings are in agreement with PAS motif distribution in other species. The remaining 29 transcripts, without any of the two most common PAS motifs, were analysed using the TEIRESIAS algorithm [38] to reveal the most frequently occurring 6-mers (see methods). This analysis showed that there was no single motif shared between all the sequences. However, the most frequent hexamers, present in five or more of the 29 transcripts (table 3), did share sequence similarity with the canonical PAS sequence. Together with their location, this could suggest these 6-mers as candidate variant PAS signals in *Salmo salar*. There was no significant difference observed when comparing the average 3'UTR length in the group of transcripts with the canonical PAS versus the group of transcripts with alternative PAS (data not shown).

**Table 3:** Most common hexamers^1^

**Sequence**	**No^2^**
UAUAAA	7
AAUAUA	6
AUAAAA	6
AAAAUA	5
AAAUAU	5
AAGAAA	5
AUAUAA	5

^1^Most common hexamers within 35

nucletides upstream of the poly-A tail

^2^Number of transcripts with this motif

### Conserved candidate regulatory motifs in *Salmo salar *transcripts

UTRs are usually rich in motifs (target sequences) that contribute in modification of gene expression by binding proteins or microRNAs (miRNAs). The UTRs from this study provides a first opportunity to identify candidate functional motifs in salmon transcripts. To find such motifs we searched the salmon UTRs for matches to evolutionary conserved motifs. The UTR database collection (UTRdb) is a collection of functional sequence patterns located in 5' or 3' UTR sequences [39]. By use of the pattern matcher program UTRscan we searched the UTRdb using the 5' and 3'UTRs as queries to identify putative regulatory motifs in the salmon transcripts. The analysis of the 5'UTRs revealed two transcripts [GenBank: BT043818 and BT043819] with motifs that were matches to the iron responsive element (IRE). IRE is a particular hairpin structure located in 5'UTRs of mRNAs involved in regulation of cellular iron metabolism [40]. The salmon transcripts were annotated as two variants of the ferritin heavy polypeptide. The observation that this motif, known to be evolutionary conserved and present in the ferritin genes of vertebrates [41], were located in the 5'UTR of these salmon ferritin transcripts supports that the matches were not by chance, but that the motifs are true functional IREs. In the analysis of 3'UTRs one transcript with a sequence that matched the SECIS element was revealed. The SECIS element is a 60 bp stem-loop structure located in the 3'UTR that directs the cell to translate the UGA codons as selenocystein instead of terminating the translation [42]. The finding that the salmon transcript with the match to this motif encodes a glutathione peroxidase [GenBank: BT044014], a gene that in other species has been shown to be a selenium-containing protein that translate UGA codons, supports the fact that also this motif match reveals a true functional element.

MicroRNAs (miRNAs) are a family of 21-25 nucleotides small RNAs. The first 1-7 or 2-8 nucleotides of the miRNA (the seed) binds to a 3'UTR target in a sequence specific manner. When base paired to its target sequence the miRNA down regulates gene expression of the target transcript. Genes that are regulated by miRNAs have been identified in vertebrates, plants, flies and worms [43,44]. It would be expected that gene expression is modified by miRNAs in salmon as well, but as far as we know, no miRNAs or their target genes have so far been described in salmon. A common feature of miRNAs is that one miRNA often regulates the expression of many genes [44]. Thus, all genes regulated by a given miRNA share an identical (or highly similar) 7-mer miRNA target sequence. If there were one miRNA that regulate expression of a large number of genes in salmon, then the target sequence of that miRNA would be present in a larger number of 3'UTRs than expected by chance. The 3'UTRs from our material provided an opportunity to identify such miRNA target sequences. To reveal such candidate conserved motifs we first identified the most frequently occurring 7-mer motifs in the 3'UTRs using the TEIRESIAS sequence pattern discovery tool [38]. We disregarded motifs that matched the PAS (five or more matching nucleotides) or consisted of simple sequence, and were left with 11 motifs that were present in more than 60 of the 3'UTR sequences. If the sequences from all 3'UTRs were pooled the size would be 226,643 bp. A 7-mer motif is expected to occur about 14 times by chance in a fragment of that size. This expectation is based on assumptions like an equal proportion of all four nucleotides and do not take into account that some sequences are over-represented cause they originate from interspersed repeats. Thus, to better evaluate whether these motifs were conserved in the 3'UTRs we compared the number of times a given motif was observed in the 3'UTRs to the number of times these motifs were observed in three control sequences. A salmon BAC sequence [GenBank: DQ156149] consisting of 219,899 bp genomic DNA was used as one such control. We did notice that the nucleotide composition in the UTRs and the BAC salmon sequence was slightly different. In the 3'UTRs the proportions were A (29%), G (19%), C (20%), T (32%) while the proportions in the genomic sequence was A (29%), G (21%), C (22%), T (28%). Thus, to have a control with an identical nucleotide composition as in the 3'UTRs we pooled the 3'UTR sequences, created ten randomly shuffled references, and counted the number of times the motifs were present in each of these. Finally, the reverse 3'UTR strands were used as a third control group. An over-representation of a certain motif in the 3'UTRs when compared to the reverse strand could indicate a strand asymmetry in distribution of this motif. The sequences of the eleven motifs, the number of times a given motif was observed in the 3'UTRs and in the three controls as well as p-values from comparison with the random shuffle control sequences are given in table 4. The comparisons showed that each of the different eleven 7-mers were about twice as frequent in 3'UTRs, and statistical tests (see methods) showed a significant over-representation of all motifs in the 3'UTRs.

**Table 4:** Conserved motifs in salmon 3'UTRs

**No**	**Motifs^1^**	**Observed^2^**	**Genomic^3^**	**Random seq^4^**	**Reverse^5^**	**p-value^6^**	**miRNA/protein^7^**
1	ATGTTTT	127, 109	48	39 +/- 6	55	39.2	
2	AATGTTT	118, 99	40	36 +/- 4	57	34.4	miR-543
3	TACTGTA	90, 85	31	20 +/- 6	30	45.2	miR-101, miR-199, miR-144
4	TAAATGT	80, 75	42	32 +/- 4	37	19.4	
5	ATGTATT	89, 73	27	34 +/- 5	34	16.1	miR-466b-3-3p
6	TGTAAAT	79, 72	39	33 +/- 5	30	16.2	PUF
7	TTGTAAA	79, 68	29	31 +/- 6	46	15.4	PUF
8	TTGTATT	70, 60	23	37 +/- 7	28	6.0	CFI
9	AATGTAT	83, 64	28	28 +/- 4	33	15.6	CFI
10	TGTCTGT	76, 63	27	16 +/- 6	41	30.6	
11	TGTCATT	70, 61	37	24 +/- 4	37	17.6	miR-425-5, miR-731, miR-489

^1^Sequence of common 7-mers in the 3'UTRs,

^2^Total number of times the motif was observed in 3'UTRs, number of 3'UTR sequences with the motif

^3^Number of times the motif was observed in salmon genomic DNA (from BAC sequence DQ156149)

^4^Mean number of times the motif was observed in ten separate shuffling trials along with standard deviation

^5^Number of times the motif was observed on the reverse strand of the 3'UTRs

^6^Chi-square p-value using a 2 × 2 contingency table as described in methods

^7^miRNAs or proteins known to bind 3'UTR target sequences identical to the motif

Conservation of particular sequences could itself indicate some kind of function. To elucidate whether any of these motifs were miRNA target sequences we compared the sequence of the eleven conserved motifs to the seven first nucleotides (the seed) of miRNAs in the miRBase as well as the seeds from miRNAs reported in a recent study of rainbow trout [44-46]. This comparison revealed that four of the motifs (motif 2,3,5 and 11 in table 4) were complementary sequences to seeds of one or more miRNAs in the miRBase. The miRNAs with complementary seeds to a given motif are listed in table 4. The finding that four of these conserved 7-mers are identical to target sequences of evolutionary conserved miRNAs further suggested that they were true functional miRNA target sequences. The putative target sequences were present in more than hundred of the 3'UTRs and if they are true target sequences, this indicates that the expression of more than 20% of the genes in this material may be regulated by miRNAs. Further experimental validation studies of these genes could confirm that they are true miRNA targets.

Two other conserved 7-mers (motif 6 and 7 in table 4) are matches to the PUF consensus sequence. The Puf family of proteins is an evolutionary conserved family of proteins that, in a similar manner to miRNAs, bind target sequences in 3'UTRs and repress gene expression [47]. The complete consensus PUF sequence is an 8-mer (TGTAHATA), and a search for this motif revealed 63 matches in the 3'UTRs. This suggests that these conserved motifs represent binding sites for homologous Puf proteins in salmon. There were five conserved 7-mers with no match to a 7-mer (or larger) sequence with a known function. We note that two 7-mers (motif 8 and 9) are matches to the shorter CFI target sequence TGTAN that participate in polyadenylation site recognition [48]. We also note that the four first bases of motif 4 in table 4 is a match to the common PAS, and in 16 sequences the motif is located in overlap with the PAS. This could possibly explain why these three motifs are conserved. The putative causes of conservation of the remaining two motifs are unknown. Experimental validation of the motifs could provide further knowledge about their function. A complete list of transcripts with the different putative target sequences is available from author upon request.

## Conclusion

The results from this study demonstrate that a large number of the full-length sequenced cDNA clones from the white muscle tissue and developmental stage specific library contained complete coding sequences. We estimated that at least 79% (440 out of 560) of the clones selected for sequencing represent transcripts with complete CDSs. This represents about a quarter of all different contigs and singlets in the library. The selection and validation methods used in this study are conservative since they filter out clones with ORFs that has not been discovered in other species. Thus, the percentage of clones with complete cCDSs in the library may be slightly larger due to the possible presence of clones with cCDSs not yet described in other species or unique to salmon. The salmon genome resources produces by the Salmon Genome Project [9,10] include another 23 tissue and developmental stage specific cDNA libraries that were constructed by use of a non full-length enriched method. We have shown by this study that, although our library was constructed with the same method such cDNA libraries represent a valuable source for attaining transcripts with complete coding sequences. This is important knowledge since the additional libraries are likely to include transcripts from genes, or particular splice variants of genes, that are tissue and/or developmental stage specific expressed.

The analysis of salmon UTRs revealed several motifs that were significantly over-represented in the 3'UTRs. The fact that a subset of these motifs was identical to miRNA target sequences suggests that they are true miRNA targets. Although further experimental validation are needed to confirm the putative function of these motifs, the results indicate that the salmon UTR sequences retrieved from full-length insert sequencing of cDNA clones may be used to identify functional motifs important in regulation of gene expression. Salmon has been suggested as a model species to study the evolution of coding sequence divergence in ohnologs. If salmon miRNAs and their target transcripts are identified the salmon may also represent a model species for studying the evolution of divergent gene expression in ohnologs.

## Methods

The cDNA clones selected for full-length sequencing were from a non full-length enriched white muscle tissue specific library constructed as described in Adzhubei et al. [10]. All 6,656 cDNA clones in this library were from the pre-smolt developmental stage. After sequence processing and clustering the 6,656 cDNA clones had been assembled in 567 contigs and 1,091 singlets [10].

### Selection of cDNA clones by homology searches in databases

To select which cDNA clones from 567 contigs and 1,091 singlets to undergo full-length sequencing we used homology analysis. Lack of similarity to the N-terminal end of proteins in the database was used as a mean for removing cDNA clones not likely to contain complete coding sequences. Consensus sequences from the contigs and the 5'EST sequences from the singlets were used as input (query) and compared to data in PDB, Swiss-Prot and nr protein sequence databases using BLASTX [24]. Threshold for a significant match was defined as expectation value < 10^-15^. Only cDNAs that contributed input-sequences providing a match to the N-terminal end of proteins in the databases were considered for full-length sequencing. In the cases where the query was a contig consensus sequence one cDNA clone contributing a sequence that aligned at the 5' end of the contig was chosen for full-length sequencing. Based on these simple selection criteria's 560 cDNA clones, 273 selected from contigs and 287 singlets, were chosen for full-length sequencing.

### DNA sequencing of cDNA clones

Sequencing was performed as described in Adzhubei et al. 2007 using T3 and -21 m13F sequencing primers for sequencing from 5' and 3' end of the transcripts, respectively [10]. Additional custom primers for primer walking were designed using MacVector software. All chromatograms were manually inspected, and sequences from individual clones were assembled into full-length contigs using Sequencher 4.7 software. The sequence data representing the CDS in each of the insert sequences were generated from at least two reads. The finished full-length sequenced inserts were covered by an average of 4.1 reads (total number of bases sequenced/total number of bases in all FLIcs).

### Characterization of coding sequences (CDSs) and UTRs

To evaluate whether a FLIc contained a complete CDS we performed BLASTX similarity searches against the RefSeq protein database [24]. Expectation value <10^-15 ^and a positive score of at least 60% were used as threshold values for a significant match. Significant matches were further validated by manual inspection of the alignments. We considered a FLIc to contain a complete CDS if the ORF from the FLIc revealed a startcodon and stopcodon in agreement with the match in the database. The start and stop codons of CDSs were used to define the boundary between the coding sequence and the 5' and 3'UTRs. If a significant match did not contain a startcodon in the 5' end of the coding sequence and the pairwise alignment indicated that the transcript lacked some 5' coding sequence it was considered to be a FLIc with a partial coding sequence.

### Gene ontology annotation

FLIcs were assigned gene ontology terms by use of Blast2GO [26]. The annotation was performed with default Blast2GO conditions.

### Comparison of cCDS FLIcs with TCs in the DFCI salmon gene index

Transcripts (two or more) that were likely to be members of the same gene family (based on CDS sequence similarity and annotation) were grouped together. There were a total of 54 such small groups in our material. Thirty-five randomly chosen FLIc from each of these small groups, not including any of the small FLIcs from groups annotated as ribosomal proteins, was used in the comparison with a "matching" (see below) tentative consensus sequence (TC) from the Dana-Farber Cancer Institute (DFCI) salmon gene index [28]. The salmon TCs in the DFCI gene index are constructed by use of a large number of ESTs (432,243 EST, October 2008) including the ESTs submitted from the Salmon Genome Project [9,10]. This allowed a comparison between the cCDS FLIcs and the TCs containing the EST from the same source clone as the cCDS FLIcs. The aligned pairs of TC sequences and cCDS FLIcs were manually inspected. All comparisons that showed four or more differences in every 100 bp of the aligned sequences were classified as non-matches.

### Search for frequently occurring motifs

Searches for most frequently occurring motifs in the UTRs were performed by use of the TEIRESIAS-based pattern discovery tool [38]. A search for frequently occurring 6-mers (putative PAS motifs) was performed in the 29 transcripts without the two most common PAS (AAUAAA, AUUAAA). The 35 bp immediate upstream of the poly A-tail from these transcripts was used as input. Pattern discovery tool conditions were: exact discovery, L = 6 and W = 6. A search for most frequently occurring 7-mer (putative target sequences) was performed in 3'UTRs from 469 FLIcs (427 cCDS FLIcs and 42 partial CDS FLIcs) with conditions: exact discovery, L = 7 and W = 7.

### Testing for statistical over-representation of motifs in 3'UTRs

To test whether the 7-mers identified by use of the TEIRESIAS-based pattern discovery tool were significantly over-represented in 3'UTRs we compared the number of times a given motif was present at least once in a 3'UTR sequence to the number of times this motif was observed in three different kinds of reference sequences (controls). One control was a genomic DNA sequence from a salmon BAC clone [GenBank: DQ156149]. The size of this sequence was 219,899 bp. A second control was generated by pooling all 3'UTR sequences into one large continuous sequence (226,643 bp), and then create ten separate randomized controls using the shuffle DNA tool at the sequence manipulation site . The mean number of occurrences of each of the motifs from these ten shufflings was used for comparison. A third control was the reverse strand of all 3'UTR sequences. The significance of the difference in number of a given 7-mer observed between 3'UTRs and the "random shuffle control" was examined using a 2 × 2 contingency table. A chi-square test was used to test the null hypothesis that motifs were evenly distributed among the two groups. There are more intricate methods to test for significant over-representation of motifs [49]. However, since we revealed a significant difference between 3'UTRs and the control in all eleven cases using the simple method described here, more advanced methods was not applied.

### Identification of miRNAs with "seeds" that was complementary to conserved 3'UTR motifs

The reverse complementary sequence of the eleven most common 7-mer motifs in the 3'UTRs was compared to the seed region (first seven bases in the miRNAs) of known miRNAs. The miRNA sequences were from the miRBase version 5 . Only perfect matches to miRNA seed regions were reported in table 4.

## Authors' contributions

RA participated in sequencing of FLIcs, performed GO annotation of FLIcs, loaded all sequences to GenBank, carried out bioinformatics and statistics, and drafted the manuscript.

BH conceived, designed and coordinated the study, constructed and performed the initial sequencing of the cDNA library and helped draft the final manuscript.

SL performed major part of the FLIcs sequencing and manual processing of the data.

All authors have read and approved the final manuscript.

## Supplementary Material

Additional file 1 Contains complete dataset from cCDS FLIcs with GenBank accession numbers, sequence descriptions, cDNA clone reference numbers, GO terms and enzyme codes. Click here for file

Additional file 2 Contains complete dataset from the partial CDS FLIcs with GenBank accession numbers, sequence descriptions, cDNA clone reference numbers, GO terms and enzyme codes. Click here for file

Additional file 3 Contains a table that shows the groups of putative paralogs including their GenBank accession numbers and sequence descriptions. Click here for file
